# Cross-national comparisons of adolescent mental health and suicidality in the Middle East: a cross-sectional secondary analysis of the global school-based student health survey

**DOI:** 10.3389/fpsyt.2026.1865386

**Published:** 2026-07-24

**Authors:** Atika Khalaf, Basma Al Yazeedi, Anas Alsharawneh

**Affiliations:** 1 The PRO-CARE Group, Faculty of Health Science, Kristianstad University, Kristianstad, Sweden; 2 Hind Bint Maktoum College of Nursing and Midwifery, Mohammed Bin Rashid University of Medicine and Health Sciences, Dubai Health, Dubai, United Arab Emirates; 3 College of Nursing, Sultan Qaboos University, Muscat, Oman; 4 Department of Adult Health Nursing, Faculty of Nursing, The Hashemite University, Zarqa, Jordan

**Keywords:** adolescents, GSHS, mental health, Middle East, protective factors, suicidality

## Abstract

**Background:**

Adolescent mental health and suicidality represent growing global public health concerns, yet comparative evidence from the Middle East remains limited. This study examined the prevalence and correlates of psychological distress and suicidal behaviors among adolescents across Middle Eastern countries using the Global School-based Student Health Survey (GSHS).

**Methods:**

A cross-sectional analysis of nationally representative GSHS datasets from 12 Middle Eastern countries collected between 2004 and 2017. The pooled sample included 90,573 school-attending adolescents aged 11–18 years. Multivariable logistic regression models assessed associations between psychosocial factors and suicidal behaviors while adjusting for age, sex, and country.

**Results:**

Substantial cross-country variation in mental health indicators and suicidality was observed. Loneliness ranged from 11.5% in Bahrain to over 50.0% among female adolescents in Tunisia and Morocco. Suicidal ideation affected 16.3% of adolescents overall, while suicide planning was reported by 13.4%. Suicide attempts were reported by 9.1% of adolescents at least once. Female adolescents consistently reported higher levels of loneliness and anxiety, whereas males showed slightly higher adjusted odds of suicidal ideation and attempts (OR = 1.2). Lack of parental support, low school connectedness, and fewer friendships were significantly associated with higher odds of suicidal ideation, planning, and attempts. Emotional symptoms such as loneliness (OR = 1.7) and anxiety (OR = 1.8) were also strongly associated with suicide attempts.

**Conclusions:**

Psychological distress and suicidality are prevalent among adolescents across the Middle East, with notable gender and cross-country differences. Strengthening family support, peer relationships, and school connectedness should be central to regional adolescent mental health and suicide prevention strategies.

## Introduction

Adolescence is a vulnerable period for the onset of mental health problems, with the most comprehensive meta-analysis (192 studies; n≈709k) estimates ~35% of first onsets occur before 14, ~48% before 18, and ~63% before 25 (peak onset ≈14.5 years) (1). Common mental health challenges among adolescents include psychological distress, loneliness, anxiety, and suicidal ideation or attempts, all of which are strongly associated with adverse life outcomes (2, 3). Globally, suicide remains a leading cause of death among young people, accounting for a significant proportion of preventable mortality (4). Suicide is the third leading cause of death among people aged 15–29 years worldwide, and recent Global Burden of Disease estimates highlight its ongoing impact and the fact that many of these deaths are preventable (4, 5).

Despite its importance, adolescent mental health often receives less attention than other public health priorities, particularly in low- and middle-income countries (LMIC) (6, 7). UNICEF’s *State of the World’s Children 2021* argues that adolescent mental health has been “ignored for too long,” (6). Recent commentaries and reviews highlight data and service gaps in LMICs and the scarcity of cross-national adolescent mental-health research that actually includes LMICs (7).

Across the Middle East, adolescents face distinctive psychosocial stressors related to armed conflict, displacement, economic hardship, and/or stigma, which are reflected in high rates of psychological distress and suicidality (8–10). Studies from conflict-affected settings and refugee populations report substantial burdens of post-traumatic stress disorder and depression (11, 12), while school-based surveys from countries such as Palestine (13), Lebanon (14), Jordan (15), and the United Arab Emirates (UAE) (16) indicate that roughly 10-20% of adolescents report suicidal ideation and notable proportions report suicide attempts, with Eastern Mediterranean estimates among the highest across WHO regions (17). Common correlates of suicidality include loneliness, anxiety, bullying, violence exposure, food insecurity, substance use, and low parental support (8, 18). However, most evidence comes from single-country, cross-sectional studies or specific conflict-affected groups, so multi-country analyses capturing the broader Middle Eastern adolescent population remain limited (17, 19), despite the availability of standardized data through the Global School-based Student Health Survey (GSHS).

This study is guided by the socio-ecological model, which conceptualizes adolescent mental health as shaped by interacting influences across individual, interpersonal, and societal levels (20). Individual vulnerabilities such as coping capacity influence risk, while family support, peer relationships, and bullying experiences play key roles in emotional well-being. At the community level, school connectedness is protective, whereas exposure to violence and displacement increases risk. Broader societal factors, including stigma and access to mental health services, further shape outcomes. This framework supports a multi-level understanding of adolescent psychological distress and suicidality across countries.

To date, there has been no comprehensive comparative study examining mental health indicators and suicidality across Middle Eastern GSHS countries. Here, mental health indicators refer to self-reported symptoms of internalizing distress, such as loneliness and anxiety, which reflect adolescents’ emotional well-being and potential psychological difficulties. Available studies largely report prevalence estimates within single settings, without exploring regional similarities, differences, or the sociodemographic correlates of adolescent mental health. This gap may limit policymakers’ ability to design targeted and culturally sensitive interventions. This study aims to compare the prevalence and patterns of mental health indicators and suicidality among adolescents in Middle Eastern countries using GSHS data. Based on the socio-ecological framework and previous literature, we expected that lower levels of parental support, school connectedness, and peer relationships would be associated with greater odds of suicidal ideation, planning, and attempts. We also anticipated substantial cross-country variation in mental health indicators and suicidality.

## Methods

### Study design and data source

This study was a cross-sectional secondary analysis of nationally representative GSHS datasets collected between 2004 and 2017. The study included all publicly available GSHS datasets from Middle Eastern countries that met the inclusion criteria. At the time of data extraction, no more recent GSHS datasets from eligible Middle Eastern countries were available through the WHO NCD Microdata Repository beyond 2017. Because the primary objective was to compare prevalence and correlates across countries rather than estimate temporal trends, datasets from all available survey years were pooled.

The GSHS is a collaborative surveillance project led by the WHO and the U.S. Centers for Disease Control and Prevention that collects nationally representative data on health behaviors and protective factors among adolescents (21, 22). Its standardized sampling and questionnaire design allow for valid cross-national comparisons (21, 22).

### Study population

The target population consisted of school-attending adolescents aged 11–18 years in Middle Eastern countries that had implemented the GSHS within the past two decades. Countries were included if they were geographically located in the Middle East, had publicly available GSHS datasets, and had administered the mental health module containing the outcomes of interest. Participant recruitment, eligibility screening, and response-rate calculations were conducted by the original GSHS survey teams in each country. Consequently, detailed participant-level exclusion information was not consistently available across all datasets included in this secondary analysis.

### Sampling design

The GSHS employs a two-stage cluster sampling design. At the first stage, schools are selected based on the number of enrolled students. At the second stage, classes within selected schools are randomly chosen, and all students in those classes are invited to participate. The survey is self-administered, anonymous, and conducted during one class period. Sample weights are provided to adjust for nonresponse and the probability of selection, ensuring that estimates are nationally representative.

### Measures

The GSHS is a standardized, self-administered, anonymous school survey with a common methodology across countries. Its questionnaire comprises 10 core modules that cover major behavioral risk and protective factors: alcohol use, dietary behaviors, drug use, hygiene, mental health, physical activity, protective factors (e.g. parental and peer support), sexual behaviors, tobacco use, and violence and unintentional injury, from which countries select at least six, plus basic demographics (23). Age categories were selected to reflect developmental stages commonly used in adolescent health research, distinguishing early adolescence (11 years), middle adolescence (12–15 years), and late adolescence (16–18 years).

The mental health module includes mental health indicators, including self-reported loneliness and anxiety. Previous multi-country studies using GSHS data have demonstrated acceptable reliability and cross-national comparability of mental health and suicidality indicators, supporting their use in international comparative research (18, 24). Loneliness was defined as feeling lonely most of the time or always during the past 12 months, while anxiety was assessed by reporting being so worried that one could not sleep most of the time or always during the same period (25). Suicidality was evaluated using three indicators: suicidal ideation, defined as seriously considering attempting suicide in the past 12 months; suicide planning, defined as having made a plan about how to attempt suicide during the past 12 months; and suicide attempts, defined as having actually attempted suicide one or more times in the past 12 months (25). Protective factors encompassed parental support and school connectedness. Parental support was measured by reporting that parents or guardians most of the time or always understood the respondent’s problems and worries. School connectedness was assessed by reporting feeling close to people at school, or that peers were kind and helpful most of the time or always (25). Sociodemographic covariates included age, sex, and country.

### Data analysis

The analysis was conducted in several sequential stages. First, descriptive statistics were used to estimate the weighted prevalence of mental health indicators and suicidality for each country, with results stratified by sex. Second, cross-country comparisons were performed using chi-square tests and 95% confidence intervals to identify significant differences in prevalence rates among the two genders. Third, sex differences were examined by calculating prevalence ratios to compare male and female students within each country. Fourth, multivariable logistic regression models were employed to assess the associations between protective factors, such as parental support and school connectedness, and suicidal behaviors, while adjusting for sex, age, and country. The country was included as a covariate in all multivariable models to partially account for contextual differences across settings. Nevertheless, temporal heterogeneity remains a limitation, and findings should be interpreted cautiously.

All reported odds ratios should be interpreted as measures of association rather than risk estimates. Given the cross-sectional design and the prevalence of some outcomes, odds ratios may overestimate the magnitude of associations relative to risk ratios.

The analyses were conducted using the statistical software R, and significance levels were set at p ≤ 0.05. In addition, all descriptive and regression analyses incorporated the sampling weights provided within the GSHS datasets to account for the complex survey design and to generate nationally representative estimates.

### Ethical considerations

For this secondary analysis, we used anonymized, publicly available data; therefore, additional institutional review board approval was not required. However, the GSHS received ethical approval from the ministries of health and education in each participating country. Informed consent procedures varied by country but typically included parental consent and student assent.

## Results

### Study sample characteristic

Across 12 Middle Eastern countries and survey waves conducted between 2004 and 2017, the pooled sample comprised 90,573 participants. Country-specific sample sizes ranged from 2,038 in Iraq (2012) to 24,220 in the United Arab Emirates (2005, 2010, 2016). The proportion of females varied by setting, from 43.3% in Iraq to 56.2% in Lebanon; most country samples were roughly gender-balanced (48.3-52.9%). Mean age across all years and countries indicates relatively tight age dispersion within each sample (**Table 1**). Multiple countries contributed data from multiple survey waves (e.g., Jordan, Kuwait, Lebanon, Morocco, United Arab Emirates), supporting temporal and cross-country comparisons. Characteristics of the study population by country are summarized in **Table 1**.

**Table 1:** Characteristics of the study population by country and survey year (n=90573).

Country	Survey year	Sample size	Female n (%)	Mean age (SD)
Bahrain	2016	7141	3449 (48.3)	14.1 (1.6)
Iraq	2012	2038	882 (43.3)	14.3 (1.3)
Jordan	2004, 2007	2457, 2197 (total=4654)	2329 (50.0)	14.8 (0.9)
Kuwait	2011, 2015	2672, 3637 (total=6309)	3226 (51.1)	14.8 (1.4)
Lebanon	2005, 2011, 2017	2286, 5115, 5708 (total=13109)	7366 (56.2)	14.3 (1.6)
Mauritania	2010	2063	1069 (51.8)	14.7 (1.2)
Morocco	2006, 2010, 2016	2670, 2924, 2924 (total=8518)	4049 (47.5)	14.1 (1.3)
Oman	2015	3468	1781 (51.4)	15.4 (1.6)
Palestine	2010	14558	7695 (52.9)	13.9 (1.0)
Tunisia	2008	2870	1453 (50.6)	13.8 (1.2)
UAE	2005, 2010, 2016	15790, 2581, 5849 (total=24220)	12417 (51.3)	14.2 (1.5)
Yemen	2014	2655	1283 (48.3)	14.8 (1.9)
Total	2004-2017	90573	46999 (51.9)	14.4 (1.4)

UAE, United Arab Emirates.

### Prevalence of mental health indicators

Sex differences in loneliness were significant in most countries, with higher prevalence among females, except in Bahrain (male 11.5% vs female 10.9%, χ^2^=0.5, p=.443) and Mauritania (male 34.9% vs female 33.4%, χ^2^=0.5, p=.458). The lowest loneliness rates were reported among Bahraini females (10.9%) and males (11.5%), while the highest rates were observed among females in Tunisia (51.9%) and Morocco (50.2%). The prevalence of anxiety was significantly higher among females compared to males across all countries. Further details are presented in **Table 2**.

**Table 2:** Prevalence of mental health indicators, presented in row n (%), (n=90573).

Country	Sex	Loneliness n (%)	*χ2* p - value	Anxiety n (%)	*χ2* p - value
Bahrain	Male	425 (11.5)	0.5 .443	1108 (30.1)	236.5 <.001 *
	Female	378 (10.9)		1649 (47.8)	
Iraq	Male	274 (23.9)	35.4 <.001 *	244 (21.3)	60.4 <.001 *
	Female	318 (36.1)		326 (36.9)	
Jordan	Male	580 (25.5)	68.4 <.001 *	601 (26.4)	181.2 <.001 *
	Female	857 (36.8)		1059 (45.5)	
Kuwait	Male	963 (32.2)	136.5 <.001 *	1036 (34.6)	183.8 <.001 *
	Female	1506 (46.7)		1667 (51.6)	
Lebanon	Male	1289 (22.5)	326.9 <.001 *	1568 (27.4)	387.1 <.001 *
	Female	2741 (37.2)		3248 (44.1)	
Mauritania	Male	337 (34.9)	0.5 .458	235 (24.4)	5.1 .024 *
	Female	357 (33.4)		308 (28.8)	
Morocco	Male	1457 (33.5)	237.3 <.001 *	1313 (30.2)	166.2 <.001 *
	Female	2031 (50.2)		1775 (43.8)	
Oman	Male	553 (34.1)	7.8 .005 *	561 (34.5)	111.5 <.001 *
	Female	689 (38.7)		936 (52.5)	
Palestine	Male	1745 (26.4)	126.9 <.001 *	2027 (30.6)	217.0 <.001 *
	Female	2702 (35.1)		3275 (42.5)	
Tunisia	Male	488 (35.4)	78.9 <.001 *	535 (38.8)	82.2 <.001 *
	Female	755 (51.9)		811 (55.8)	
UAE	Male	3599 (31.1)	293.2 <.001 *	3304 (28.5)	561 <.001 *
	Female	5181 (41.7)		5368 (43.2)	
Yemen	Male	396 (31.8)	15.2 <.001 *	384 (30.8)	31.4 <.001 *
	Female	504 (39.3)		534 (41.6)	

* Statistically significant value.

### Suicidal ideation, plans, and attempts across countries

Across the included settings, prevalence estimates of suicidal ideation, plans, and attempts varied widely. In general, ideation exceeded planning, which in turn exceeded attempts. Suicidal ideation ranged from a rate of 4.2% in Tunisia to 21.9% in Oman and 19.9% in Palestine. Several Gulf states also showed elevated ideation, e.g., Kuwait, 18.3%. Suicide plans were generally lower than ideation yet remained substantial in several contexts, highest in Palestine (17.2%) and Iraq (16.1%), and lowest in the UAE (10.5%) and Lebanon (10.2%). One suicide attempt appeared highest in Palestine (25.1%), while Mauritania (3.6%) and Yemen (4.9%) were at the lower end. UAE (11.4%), Morocco (10.7%), Kuwait (11.1%), and Lebanon (15.5%) fell in the mid-range. Where attempt frequency data were available, most individuals reporting attempts clustered in the lower-frequency category (one attempt), with progressively fewer in higher-frequency groups, though several countries lacked complete breakdowns. Further details are presented in **Table 3**.

**Table 3:** Suicidal ideation, plans, and attempts across countries (n=90573).

Country	Suicide ideation n (%)	Suicide plan n (%)	Number of suicidal attempts (times) n (%)			
			1	2 or 3	4 or 5	≥ 6
Bahrain	953 (13.6)	951 (13.4)	538 (7.6)	155 (2.2)	79 (1.1)	50 (0.7)
Iraq	345 (17.4)	316 (16.1)	226 (11.2)	58 (2.9)	23 (1.1)	29 (1.4)
Jordan	731 (16.6)	686 (15.6)	0	0	0	0
Kuwait	1088 (18.3)	932 (15.7)	573 (11.6)	256 (4.2)	109 (1.8)	124 (2.0)
Lebanon	1903 (14.8)	1307 (10.2)	571 (11.6)	169 (2.1)	77 (1.0)	65 (0.8)
Mauritania	366 (18.2)	311 (15.6)	204 (4.2)	74 (3.7)	33 (1.6)	46 (2.3)
Morocco	1351 (16.2)	1178 (14.3)	514 (10.5)	154 (2.7)	74 (1.3)	80 (1.4)
Oman	732 (22.0)	0	0	0	0	0
Palestine	2805 (19.8)	2412 (17.2)	1622 (33.1)	687 (4.8)	294 (2.1)	259 (1.8)
Tunisia	591 (21.0)	387 (13.9)	0	0	0	0
UAE	2830 (13.3)	2209 (10.6)	438 (8.9)	142 (2.4)	97 (1.6)	55 (0.9)
Yemen	407 (16.1)	363 (14.4)	217 (8.3)	85 (3.3)	35 (1.3)	36 (1.4)
Total	14102 (16.3)	11052 (13.4)	4903 (9.1)	1780 (3.3)	821 (1.5)	744 (1.4)

### Predictors of suicidal ideation

Suicidal ideation is clustered with lack of parental support and absence of school connectedness. Adolescents reporting no parental support and those not feeling connected to school each had 20.0% higher odds of suicidal ideation compared with their supported/connected peers (both OR = 1.2, 95% CI 1.1-1.3, p <.001). A clear social-gradient was observed for friendships: relative to having ≥3 friends (reference), having 2 friends (OR = 1.3, 95% CI 1.2-1.4, p <.001), 1 friend (OR = 1.7, 95% CI 1.6-1.8, p <.01), and 0 friends (OR = 3.1, 95% CI 2.8–3.3, p <.001) were each associated with progressively higher odds of suicidal ideation. In adjusted models, males had higher odds than females (OR = 1.2, 95% CI 1.1-1.3, p <.001). Compared with children 11 years (reference), suicidal ideation odds did not differ for 12–15 years (OR = 1.1, 95% CI 0.9-1.3, p = .172) but were significantly higher among 16–18 years (OR = 1.4, 95% CI 1.2-1.7, p <.001). Category frequencies are provided in **Table 4**.

**Table 4:** Predictors of suicidal ideation and planning (n=90573).

Factor	Category	Suicide ideation n (%)		OR	95% CI	P
		Yes	No			
Parental support	Yes	4761 (5.6)	28137 (33.4)	1.2	1.1-1.3	<.001*
	No	8851 (10.5)	42422 (50.4)			
School Connectedness	Yes	5626 (6.6)	32412 (38.4)	1.2	1.1-1.3	<.001*
	No	8004 (9.5)	38247 (45.3)			
Number of Friends	0	1724 (2.0)	3604 (4.2)	3.1	2.8-3.3	<.001*
	1	2200 (2.5)	8163 (9.5)	1.7	1.6-1.8	<.001*
	2	2314 (2.7)	11248 (13.1)	1.3	1.2-1.4	<.001*
	≥ 3	7582 (8.8)	48705 (56.9)	The reference group		
Sex	Male	6271 (7.3)	34478 (40.2)	1.2	1.1-1.3	<.001*
	Female	7644 (8.9)	37349 (43.5)			
Age	11 Yrs	156 (0.2)	947 (1.1)	The reference group		
	12–15 Yrs	10690 (12.3)	57620 (66.5)	1.1	0.9-1.3	.172
	16–18 Yrs	3256 (3.7)	13954 (16.1)	1.4	1.2-1.7	<.001*

Factor	Category	Suicide planning n (%)		OR	95% CI	P
		Yes	No			
Parental support	Yes	3788 (4.7)	27594 (34.3)	1.2	1.1-1.3	<.001*
	No	6906 (8.6)	42086 (52.3)			
School Connectedness	Yes	4395 (5.4)	31579 (39.2)	1.2	1.1-1.3	<.001*
	No	6292 (7.8)	38189 (47.4)			
Number of Friends	0	1473 (1.8)	3481 (4.2)	3.5	3.2-3.7	<.001*
	1	1745 (2.1)	8114 (9.9)	1.8	1.6-1.9	<.001*
	2	1809 (2.2)	11238 (13.7)	1.3	1.2-1.4	<.001*
	≥ 3	5802 (7.1)	48035 (58.8)	The reference group		
Sex	Male	4932 (6.0)	33963 (41.5)	1.1	1.06-1.15	<.001*
	Female	5957 (7.3)	37009 (45.2)			
Age	11 Yrs	124 (0.2)	947 (1.2)	The reference group		
	12–15 Yrs	8545 (10.3)	57637 (69.7)	1.1	0.94-1.4	.196
	16–18 Yrs	2383 (2.9)	13034 (15.8)	1.4	1.1-1.7	<.001*

* Statistically significant value.

### Predictors of suicidal planning

Suicidal planning was more common among students reporting no parental support (8.6%) than those with parental support (4.7%), and among those with low school connectedness (7.8%) compared with connected peers (5.4%); both factors were significant predictors in the adjusted model (p <.001 for each). A clear dose-response was observed for number of friends: compared with having ≥3 friends (reference), having 2 friends (OR = 1.3; 95% CI: 1.2-1.4; p <.001), 1 friend (OR = 1.8; 95% CI: 1.6–1.9; p <.01), and especially 0 friends (OR = 3.5; 95% CI: 3.2-3.7; p <.001) was associated with progressively higher odds of suicidal planning. By sex, the crude prevalence was 7.3% for females and 6.0% for males, while the model indicated a small but statistically significant difference (OR = 1.1; 95% CI: 1.06-1.15; p <.001). Relative to children 11 years (reference), adolescents 16–18 years had higher odds (OR = 1.4; 95% CI: 1.1-1.7; p <.001), whereas those 12–15 years did not differ significantly (OR = 1.1; 95% CI: 0.94-1.4; p=.196). **Table 4** reports the results for predictors of suicidal planning.

### Predictors of suicide attempts

Across psychosocial and demographic factors, several variables were associated with higher odds of reporting suicide attempts. Adolescents lacking parental support and those not feeling connected to school had significantly greater odds (OR = 1.5, 95% CI: 1.3-1.6; OR = 1.4, 95% CI: 1.3-1.5, respectively; both p<.001). Having fewer friends showed a dose–response pattern compared with having ≥3 friends (reference): 0 friends (OR = 2.8, 95% CI: 2.5-3.1), 1 friend (OR = 1.7, 95% CI: 1.6-1.8), and 2 friends (OR = 1.3, 95% CI: 1.2-1.4); all p<.001. Male sex was modestly associated with higher odds versus female (OR = 1.2, 95% CI: 1.1-1.3; p<.001). Emotional symptoms were strong correlates: loneliness (OR = 1.7, 95% CI: 1.6-1.8; p<.001) and anxiety (OR = 1.8, 95% CI: 1.7-1.9; p<.001). Predictors of suicide attempts are reported in **Table 5**.

**Table 5:** Predictors of suicidal attempts (n=90573).

Factor	Category	Suicidal attemptsn (%)					OR	95% CI	*p*
		1	2	3	4	5			
Parental Support	Yes	18851 (36.1)	1418 (2.7)	439 (0.8)	235 (0.4)	191 (0.3)	1.5	1.3-1.6	<.001*
	No	25451 (48.7)	3317 (6.3)	1273 (2.4)	534 (1.0)	512 (0.9)			
School Connectedness	Yes	25125 (48.2)	2061 (3.9)	668 (1.2)	318 (0.6)	255 (0.4)	1.4	1.3-1.5	<.001*
	No	19075 (36.6)	2655 (5.1)	1028 (1.9)	453 (0.8)	435 (0.8)			
Number of Friends	0	2306 (4.3)	512 (0.9)	233 (0.4)	201 (0.3)	143 (0.2)	2.8	2.5-3.1	<.001*
	1	4757 (8.9)	819 (1.5)	300 (0.5)	132 (0.2)	105 (0.2)	1.7	1.6-1.8	<.001*
	2	6892 (13.0)	820 (1.5)	344 (0.6)	118 (0.2)	101 (0.2)	1.3	1.2-1.4	<.001*
	>= 3	31139 (58.7)	2638 (4.9)	826 (1.5)	285 (0.5)	353 (0.6)	The reference group		
Sex	Male	21434 (40.3)	2214 (4.1)	799 (1.5)	402 (0.7)	398 (0.7)	1.2	1.1-1.3	<.001*
	Female	23671 (44.5)	2611 (4.9)	937 (1.7)	396 (0.7)	318 (0.6)			
Loneliness	Yes	13043 (24.2)	2173 (4.0)	990 (1.8)	361 (0.6)	428 (0.8)	1.7	1.6-1.8	<.001*
	No	32489 (60.4)	2730 (5.1)	790 (1.4)	460 (0.8)	316 (0.6)			
Anxiety	Yes	15715 (29.2)	2462 (4.6)	1082 (2.0)	410 (0.7)	465 (0.8)	1.8	1.7-1.9	<.001*
	No	29817 (55.4)	2441 (4.5)	698 (1.3)	411 (0.7)	279 (0.5)			

Odds ratios are shown for the risk category relative to the indicated reference (≥3 friends; female; and presence of parental support and school connectedness as the low-risk references).

* Statistically significant value.

## Discussion

This cross-national study examined patterns of psychological distress and suicidality among adolescents across Middle Eastern countries using data from the GSHS. Several key findings emerged. First, substantial cross-country variation was observed in the prevalence of loneliness, anxiety, suicidal ideation, planning, and attempts. Second, female adolescents consistently reported higher levels of emotional distress, particularly loneliness and anxiety, whereas males exhibited higher adjusted odds of suicidal ideation and attempts. Third, social relationships, including parental support, school connectedness, and peer friendships, were strongly associated with suicidal behaviors. Finally, emotional symptoms such as loneliness and anxiety were significant correlates of suicide attempts. From a socio-ecological perspective, these findings suggest that adolescent suicidality reflects the interaction of individual emotional vulnerabilities with interpersonal relationships and broader contextual influences across countries.

The prevalence estimates reported in this study align with global evidence showing that suicidal ideation and attempts are common among adolescents worldwide. Large, pooled analyses of GSHS data from multiple countries have similarly documented considerable variation in adolescent suicidality across regions, reflecting the influence of social, cultural, and structural determinants (18, 24). Our findings indicate that suicidal ideation affected a substantial proportion of adolescents in several Middle Eastern countries, with particularly elevated prevalence in settings such as Oman, Palestine, and Kuwait. These differences likely reflect contextual factors, including political instability, exposure to conflict, economic uncertainty, and variations in access to mental health services across the region (17, 26). Adolescents in conflict-affected contexts may experience chronic stress, displacement, and trauma, which have been repeatedly linked to higher risks of depression, anxiety, and suicidal behavior (11, 27). Within the socio-ecological framework, these patterns highlight the influence of macrosystem factors, such as political instability and health system capacity, on adolescent mental health outcomes, which calls for coordinated policy efforts to strengthen adolescent mental health services, integrate psychosocial support within school and primary healthcare systems, and prioritize regionally informed research that examines how structural and contextual determinants shape youth mental health across Middle Eastern settings.

Consistent with previous research, female adolescents in this study reported significantly higher levels of loneliness and anxiety across nearly all countries. Gender differences in internalizing symptoms during adolescence are widely documented and are often attributed to biological changes during puberty, gendered socialization processes, and differential exposure to psychosocial stressors (2, 28). However, despite higher levels of emotional distress among females, males in the adjusted models demonstrated greater odds of suicidal ideation and attempts. This pattern reflects the well-recognized “gender paradox” in suicidal behavior, whereby females report higher levels of psychological distress and suicidal thoughts, while males exhibit higher rates of suicidal behaviors and mortality (4, 29). These findings illustrate how individual biological and psychological factors interact with gendered social norms, representing both individual and societal layers of the socio-ecological model. Furthermore, cultural expectations surrounding masculinity, reduced help-seeking behaviors among males, and the use of more lethal coping strategies may partly explain these differences (30).

Social relationships emerged as a central determinant of adolescent mental health in this study. Adolescents who reported low parental support or weak school connectedness were significantly more likely to report suicidal ideation, planning, and attempts. These findings align with extensive evidence indicating that supportive family relationships and a strong sense of belonging in the school environment are key protective factors for adolescent mental health (31, 32). Family support may be associated with lower levels of emotional distress by providing emotional guidance, monitoring, and reassurance during developmental transitions. Similarly, school connectedness has been associated with lower levels of depression, suicidal ideation, and risk behaviors among adolescents in multiple international studies (33, 34). Within the socio-ecological framework, these results emphasize the critical role of interpersonal systems, particularly family and school environments, in shaping adolescent mental health outcomes, calling for the implementation of school-based mental health programs and family-centered interventions that strengthen supportive relationships and promote early identification of psychological distress among adolescents.

Peer relationships also demonstrated a pronounced dose-response association with suicidality in this study. Adolescents reporting fewer friendships, particularly those with no close friends, had substantially higher odds of suicidal ideation and planning compared with those reporting multiple friends. Social isolation and perceived lack of belonging are central components of the interpersonal theory of suicide, which posits that suicidal ideation arises when individuals experience both thwarted belongingness and perceived burdensomeness (35). Empirical evidence consistently supports the role of peer connectedness in protecting adolescents from mental health difficulties, while social isolation has been associated with increased risks of depression, anxiety, and suicidal behaviors (36). These findings further highlight the importance of peer networks as a key interpersonal layer within the socio-ecological framework influencing adolescent well-being.

Emotional symptoms were also strongly associated with suicidal behaviors in the present study. Adolescents who reported loneliness or anxiety had significantly higher odds of suicide attempts. Loneliness and chronic worry represent core indicators of psychological distress and have been linked to increased risk of self-harm and suicidal behavior among young people across diverse cultural settings (2, 18). These findings highlight the importance of early identification and intervention for emotional symptoms during adolescence, as untreated distress has been linked to more severe mental-health outcomes. In addition, these emotional symptoms represent individual-level risk factors that may be exacerbated or mitigated by surrounding interpersonal and environmental contexts (20).

Within the Middle Eastern context, broader sociocultural factors may also influence adolescent mental health outcomes. Mental illness remains highly stigmatized in many societies across the region, which can limit adolescents’ willingness to seek psychological support or disclose emotional distress (37). At the same time, strong family cohesion and community networks, characteristic of many Middle Eastern cultures, may function as important protective resources when supportive relationships are present (39). Religious and spiritual beliefs may further provide coping mechanisms that help some adolescents manage psychological distress and suicidal thoughts (38). While these contextual factors operate at the societal and cultural levels, shaping norms, stigma, and access to support systems, they underscore the importance of culturally sensitive mental-health promotion strategies in the region.

This study’s findings not only support the socio-ecological framework of adolescent mental health but also highlight the interplay between individual, interpersonal, and societal influences on adolescent psychological well-being. Moreover, emotional vulnerabilities such as loneliness and anxiety operate alongside relational factors, including family support, peer relationships, and school environments, in shaping adolescents’ mental well-being. Therefore, addressing adolescent mental health requires coordinated interventions across multiple levels of influence.

### Strengths and limitations

This study provides one of the few cross-national analyses of adolescent mental health and suicidality in the Middle East, using standardized data from the GSHS. The large, pooled sample of adolescents across multiple countries enhances statistical power and enables meaningful regional comparisons. The use of nationally representative datasets and standardized measures strengthens the reliability of prevalence estimates and cross-country comparisons (18, 24).

However, several limitations should be considered. The cross-sectional design prevents causal inference between psychosocial factors and suicidal behaviors. Additionally, the study relies on self-reported measures, which may be affected by recall bias or underreporting due to stigma surrounding mental health and suicide (17). Consequently, prevalence estimates may be conservative. Furthermore, the GSHS includes only school-attending adolescents, potentially excluding more vulnerable youth who are not enrolled in school. In addition, survey years differed across countries, which may affect comparability due to changing social, political, economic, and technological contexts. The rapid expansion of digital technology and social-media use across the study period may have influenced adolescents’ perceptions of mental health, help-seeking behaviors, and reporting of suicidal thoughts and behaviors. Furthermore, although the GSHS uses standardized instruments and procedures across countries, cultural differences in the interpretation and reporting of mental health symptoms and suicidal behaviors may still have influenced cross-country comparisons.

Although country was included as a covariate in all regression models, residual clustering within countries may remain. Future studies using multilevel modelling approaches could provide more precise estimates of contextual effects.

### Policy and public health implications

The findings highlight the need to prioritize adolescent mental health within national public health strategies in the Middle East. Schools may serve as key platforms for prevention, as stronger school connectedness was associated with lower odds of suicidality. Evidence suggests that school-based mental health programs and peer-support initiatives can reduce psychological distress and suicidal behaviors among adolescents (32, 33). Strengthening family support and parent-adolescent communication may also be important protective strategies, as supportive parenting has been consistently linked to better mental health outcomes among adolescents (31).

### Future research

Future studies should use longitudinal designs to clarify causal relationships between psychosocial factors and suicidality during adolescence. Research should also explore the role of contextual determinants, including conflict exposure, displacement, and access to mental health services, which may contribute to the cross-country differences observed in this study (27). Evaluations of culturally appropriate school- and community-based interventions are also needed to guide regional suicide prevention strategies.

## Conclusions

Adolescent psychological distress and suicidality represent significant public health concerns across Middle Eastern countries. This study demonstrates that loneliness, anxiety, and suicidal behaviors are prevalent among adolescents in the region, with notable differences across countries and between genders. Social relationships, including parental support, school connectedness, and peer friendships, emerged as key protective factors, while emotional symptoms such as loneliness and anxiety were strongly associated with suicide attempts.

These findings highlight the importance of strengthening supportive family, school, and peer environments as part of comprehensive adolescent mental health strategies. Addressing adolescent mental health in the Middle East will require coordinated efforts that integrate prevention, early identification, and accessible support services within schools, families, and health systems. Expanding research and policy attention to adolescent mental health is essential to reduce the burden of psychological distress and prevent suicide among young people in the region.

## Data Availability

Publicly available datasets were analyzed in this study. This data can be found here: https://www.who.int/teams/noncommunicable-diseases/surveillance/systems-tools/global-school-based-student-health-survey.
